# Food choice motivations and perceptions of healthy eating: a cross-sectional study among consumers in the UAE

**DOI:** 10.1186/s12889-024-20836-8

**Published:** 2025-02-04

**Authors:** Leila Cheikh Ismail, Tareq M. Osaili, Reyad Shaker Obaid, Mona Hashim, Marya Ahmed, Fatma Al-Fayadh, Aisha Farah, Hanin Sad, Humood Alghanem, Maysm N. Mohamad, Sheima T. Saleh, Rameez Al Daour, Emad Masuadi, Lily Stojanovska, Habiba I. Ali, Ayesha S. Al Dhaheri

**Affiliations:** 1 Department of Clinical Nutrition and Dietetics, College of Health Sciences, University of Sharjah, Sharjah, 27272 UAE; 2 Nuffield Department of Women’s & Reproductive Health, University of Oxford, Oxford, OX1 2JD UK; 3 Department of Nutrition and Food Technology, Faculty of Agriculture, Jordan University of Science and Technology, Irbid, 22110 Jordan; 4 Department of Nutrition and Health, College of Medicine and Health Sciences, United Arab Emirates University, Al Ain, 15551 UAE; 5 Department of Public Health Institute, College of Medicine and Health Sciences, United Arab Emirates University, Al Ain, 15551 UAE; 6 Institute for Health and Sport, Victoria University, Melbourne, VIC 3011 Australia

**Keywords:** Healthy food choices, Food selection, Dietary behaviour, Perceptions, Motivations

## Abstract

**Background:**

Investigating consumer food choice motivations is crucial for planning effective policies and targeted interventions. This study aimed to examine the food choice motivations and perceptions of healthy eating among adults in the United Arab Emirates (UAE) and to segment consumers based on their motivations.

**Methods:**

A web-based, cross-sectional study was conducted among adults in the UAE (*n* = 1209). An overall perception of healthy eating score was calculated based on the sum of the responses to the perception statements. Food motivation scores were calculated with a higher score indicating more influence of the food motivation group. Hierarchical Cluster Analysis (HCA) and K-means cluster analysis were used to identify and determine the optimal number of clusters. Differences between clusters were evaluated using an Independent sample t-test, One-Way ANOVA test, and Chi-square analysis.

**Results:**

Participants mostly agreed that a healthy diet should be balanced, varied, and complete (84.4%), that fruit and vegetables are essential to a practice of healthy eating (82.8%), and that they can eat everything as long as it is in small quantities (60.1%). Females, younger adults, those with higher education levels, and those with normal BMI tended to have a slightly more positive perception of a healthy diet than their counterparts (*p* < 0.05). Health motivation (mean = 3.43, SD ± 0.78) exhibited the highest influence on the participants’ food choices, followed by emotional motivations (mean = 3.26, SD ± 0.68). Health-related motivations mainly influenced food choices among participants in both identified clusters but were notably more emphasised in Cluster 1. Cluster 1 demonstrated significantly higher mean scores in all other categories than Cluster 2 (*p* < 0.001). Cluster 1 encompasses more female participants than males, while Cluster 2 comprises a more significant proportion of males and individuals falling within higher income brackets (*p* < 0.001).

**Conclusions:**

The results of the current study offer valuable insights into various crucial aspects that impact the decisions of individuals' food choices. Based on distinct motivational structures identified through cluster analysis, personalised approaches can encourage healthier dietary practices. A holistic approach acknowledging emotional, economic, environmental, alongside health-related factors is vital.

**Supplementary Information:**

The online version contains supplementary material available at 10.1186/s12889-024-20836-8.

## Background

Pursuing healthy and sustainable eating is a fundamental aspect of the global public health agenda [1, 2]. The dietary landscape of people is a result of the food choices they make, which can be influenced by many factors, including the proportion of food, variety, availability, and consumption frequency, among others [3]. These dietary decisions can also reflect people’s cultural and societal influences and significantly impact their health and well-being [4, 5].

In modern times, widespread consumption of unhealthy food is a significant contributor to the rising burden of chronic illnesses like obesity, diabetes, and cardiovascular disease [6]. In many countries, traditional foods are generally considered more wholesome, natural, and nutritionally rich. However, these traditional dietary patterns have been replaced by a more Westernized diet, which usually includes more processed foods loaded with sugars, unhealthy fats, animal-derived products, and refined grains [5].

In particular, there has been a significant increase in the availability of highly processed and calorie-dense foods. This shift has been driven by economic changes worldwide, which have increased people's purchasing power and made food more accessible. The rapid growth of supermarkets and the fast-food industry has further fuelled this trend, altering global eating habits [7, 8]. These readily available, heavily marketed, and affordable processed meals have flooded the food supply, surpassing actual needs [8]. Moreover, the lack of healthier options encourages a diet packed with calories, fats, and sugars, leading to weight gain and obesity [9]. Furthermore, technological advancements, particularly among younger generations, coupled with increasingly sedentary lifestyles, have also played a role in this concerning trend [10].

People's decisions about what they eat are multifaceted and constantly evolving. Key determinants of general food choices are identified and classified into several categories, encompassing factors related to the food itself (sensory and perceptual characteristics), external factors (information, social and physical environment), personal factors (biological traits, physiological needs, psychological aspects, habits, and experiences), cognitive factors (knowledge, skills, attitudes, preferences, expected outcomes, and personal identity), as well as sociocultural factors (cultural influences, economic conditions, and political factors) [3]. In other words, food choices can be influenced by people’s unique values, shaped by an individual’s life experiences and societal, environmental, and personal elements [11]. These values can be a mixture of brain-processed attributes and can be referred to as food choice motives, which can have varying levels of importance during decision-making [12]. Ferrão and Guiné et al. have developed and validated a tool that evaluates various influences on individuals' food decisions, encompassing health, emotions, cost and accessibility, societal and cultural aspects, environmental and political factors, as well as marketing and advertising [13, 14]. The Eating Motivation Scale (EATMOT) has also been validated in 16 countries and can be used to understand food choice factors across various geographical locations [15].

Understanding the motives behind food choices and advocating for a positive change is essential for reshaping the existing food system to promote individuals' well-being and the planet's sustainability [16, 17]. Policies and community-based intervention can be pivotal in realigning people's food choices with aspirations for a healthier lifestyle. Traditional evidence-based approaches to enhance public health concentrate on identifying environmental factors that result in adverse health consequences. They also focus on crafting interventions or policies that reduce exposure to these risks [18]. While several countries, including the United Arab Emirates (UAE), have been advocating and implementing changes towards healthier lifestyles, the prevalence of obesity and other non-communicable diseases remains a significant concern [19]. According to the UAE Ministry of Health, the prevalence of overweight among adults was 67.9% in 2017–2018, while those living with obesity were 27.8% [20].

The UAE has taken significant measures to promote healthy lifestyles and combat chronic diseases through initiatives like the 'Healthy Food School Box’ [21], initiating weight screening programs in schools [22], and the 'Dubai Fitness Challenge’ encouraging people to be more active [23]. The country has also imposed taxes on energy and carbonated drinks and implemented nutritional labeling policies to address the obesity pandemic [24, 25]. Despite these efforts, the prevalence of overweight and obesity remains high among different age groups [26–28], and there is still limited understanding of the factors that motivate people's food choices.

Recognizing the importance of investigating consumer food choice motivations to facilitate planning effective policies and targeted interventions, this study aimed to examine the food choice motivations and perceptions of healthy eating among adults in the UAE and to segment consumers based on their food choice motivations.

## Methods

### Study design and participants

A web-based cross-sectional study was conducted among adults in the UAE between February and May 2023. The inclusion criteria were adults (≥ 18 years – 65 years old) currently living in the UAE and with no reported history of eating disorders. These criteria were chosen as they align with the study’s aim and guarantee the appropriate use of the survey questionnaire, previously validated on adults [15]. Participants were recruited through convenience sampling, given the web-based nature of the study, and to ensure a wide distribution. The following formula was used to calculate the needed minimum sample size with a 95% confidence interval:

$$N={z}^{2}\times P\times (1-P)/{e}^{2}$$

Where z = 1.96; P = (estimated proportion of the population that presents the characteristic) = 0.5; e (margin of error) = 0.05; N (sample size) = 384 participants, plus 20% (attrition rate) = 460 participants. The survey was designed using Google Forms, and the web link was distributed through various social media platforms, including LinkedIn™, Facebook™, and WhatsApp™, leveraging the networks of the research team to reach a broad audience. To enhance participation, respondents were encouraged to share the survey link with others within their social circles. This approach successfully garnered responses from 1,209 adults, surpassing the anticipated sample size and thereby increasing the study's statistical power and enhancing the external validity of the findings.

Upon clicking on the link, participants were provided with a brief information sheet explaining the study’s objectives, protocol, and the anonymity and voluntary nature of the study. Several screening questions were also asked to ensure that participants met the inclusion criteria. Participants can then choose their language (Arabic or English) and they were then provided with an electronic consent form, and consenting participants were directed to the survey questions. The full survey is provided in the supplementary files (additional file 1).

The study received ethical approval from the Research Ethics Committee at the University of Sharjah (UOS) and adhered to the ethical standards set out in the 1964 Helsinki Declaration. To ensure the confidentiality and anonymity of participants, no identifying information was collected. Electronic informed consent was obtained from all participants before taking part in the study.

### Data collection

A self-administered, structured, web-based questionnaire was employed in this study. The survey consisted of four main sections covering socio-demographic data, perceptions of healthy food, sources of information about healthy diets, and the EATMOT questionnaire [15]. The sociodemographic section inquired about participants’ biological sex, age, city/emirate of residence, nationality, marital status, education level, employment status, and monthly household income. Participants were also asked to report their height (cm) and current weight (kg). The perceptions of healthy food and sources of information about healthy diets were adapted from a previously published article [29]. Participants were asked to rate their level of agreement for nine statements about their perception of healthy eating using a 5-point Likert scale ranging from (1) strongly disagree to (5) strongly agree. In addition, they were asked to report the frequency of using different sources of information about healthy eating (response options: (1) never, (2) rarely, (3) sometimes, (4) often, and (5) always).

The last section included the EATMOT questionnaire [15], which included a total of 49 statements asking participants about what motivates them to eat, with answers rated on a 5-point Likert scale ranging from (1) strongly disagree to (5) strongly agree. The statements were based on six different food choice motivations: (1) health, (2) emotional, (3) economic and availability, (4) social and cultural, (5) environmental and political, and (6) marketing and commercial. The items were administered randomly to participants without identification of the different groups of food choice motivations. The original EATMOT questionnaire was developed in Portuguese [14] and translated into English [15]. For this research, the questionnaire was translated into the Arabic language.

The questionnaire was translated into Arabic using a precise methodology that involved two certified translators and a back-translation by bilingual researchers [30]. A thorough review from both the research team and a panel of field experts followed this, and the authors approved the final translation. The expert panel consisted of five members: two nutrition experts, two food science experts, and one public health expert, each bringing specialized knowledge relevant to the study's focus areas. To ensure the translated questionnaire was valid, the expert panel evaluated each item on a four-part Likert scale to assess simplicity, clarity, and relevance [31]. The results showed that the questionnaire had high content validity, with an Item-Level Content Validity Index (I-CVI) ranging from 0.96 to 1.0. The Scale-Level Content Validity Index (S-CVI/Ave) was 0.99, indicating that the panel unanimously acknowledged 99% of the questionnaire's items as simple, clear, and relevant. A pilot test was then conducted on 30 participants to ensure clarity of the survey questionnaire and the data was not included in the study's final analysis.

### Statistical analysis

Data was analysed using SPSS software, version 26.0 (SPSS, Chicago, IL, USA). Continuous data were expressed as means ± standard deviations (SD), and categorical data were expressed as counts and percentages. Body mass index (BMI) was calculated by dividing participants’ self-reported weight in kilograms by the square of their height in meters. Based on the World Health Organization cut-off points, participants’ body weight status was categorized as underweight (BMI ≤ 18.5 kg/m^2^), normal weight (BMI 18.5- < 25 kg/m^2^), overweight (BMI 25- < 30 kg/m^2^), or obese (BMI ≥ 30 kg/m^2^) [32]. An overall perception of healthy eating score was calculated based on the sum of the responses of the nine statements (range 9–45). An inverted scale was used for the following items: “I believe that organic food is healthier”, “We should never consume sugary products”, and “We should never consume fat products” so that a higher score reflects a more positive perception of healthy eating. Prior to conducting the ANOVA, the data was tested for normality using the Shapiro–Wilk test. An independent sample t-test and a one-way ANOVA test were used to determine significant differences between different groups, perception statements, and food choice motivation. A Tukey post hoc test was used to identify which groups differ significantly.

Food motivation scores were calculated based on the average responses for all statements in each food motivation group, with a higher score indicating a stronger influence of the food motivation group. An inverted scale was used for negatively worded statements; “There are some foods that I consume regularly even if they may raise my cholesterol”, “There are some foods that I consume regularly even if they may raise my blood glycaemia”, “I prefer to eat alone”, “When I buy food I usually do not care about the marketing campaigns happening in the shop”, and “When I go shopping I prefer to read food labels instead of believing in advertising campaigns”. Cronbach’s alpha was used to test the internal validity of the food motivation items (49 items) (Cronbach’s α = 0.94). The food choice motivation data was used to identify consumer groups. The data included 49 items divided into six categories: health (10 items), emotional (9 items), economic and availability (7 items), social and cultural (9 items), environmental and political (7 items), and marketing and commercial (7 items). Hierarchical Cluster Analysis (HCA) was used to determine the optimal number of clusters based on Squared Euclidean distances and Ward's method. The analysis supported the formation of two distinct clusters. K-means cluster analysis was then used to confirm these clusters further. Differences between the resulting clusters were evaluated using an Independent sample t-test, One-Way ANOVA test, and Chi-square analysis. P values at < 0.05 were considered statistically significant.

## Results

### Characteristics of the study population

Table 1 presents the sociodemographic characteristics of study participants. A total of 1209 UAE residents participated in this study; 53.6% and 46.4% were females and males, respectively. Around half of the participants (43.6%) were 18–24 years old. About two-thirds of the participants were from non-GCC Arab countries (59.1%), and a similar proportion were single (61.3%). Around half of the participants (55.6%) held a bachelor's degree and were employed (49.1%). Participants had similar distributions based on income level. Moreover, based on self-reported weight and height, most of the respondents (47.7%) were of normal weight and overweight (32.2%).

**Table 1:** Sociodemographic characteristics and differences in perception of healthy eating of participants (*n* = 1209)

Characteristics	Total		Overall perception of healthy eating score		*p*-value*
	***n***	%	Mean	SD	
**Sex**					
Female	648	53.6	31.1	3.8	0.002
Male	561	46.4	30.4	4.2	
**Age (years)**					
18–24	527	43.6	31.2	4.2	** < 0.001 **^**a**^
25–29	172	14.2	31.2	3.7	
30–39	204	16.9	29.7	4.1	
40 −65	306	25.3	30.5	3.5	
**Nationality**					
GCC countries	337	27.9	30.5	3.8	0.259
Arab (other countries)	714	59.1	30.9	4.0	
Non-Arab	158	13.1	30.9	4.3	
**Marital status**					
Single	741	61.3	31.1	4.1	**0.001**
Married	468	38.7	30.3	3.7	
**Education level**					
High school or below	239	19.8	30.5	4.2	** < 0.001**^**b**^
College certificate or Diploma	187	15.5	29.7	4.3	
Bachelor’s degree	672	55.6	31.1	3.7	
Postgraduate education	111	9.2	31.6	4.0	
**Employment status**					
Employed	594	49.1	30.5	4.1	**0.011 **^**c**^
Unemployed	220	18.2	30.6	3.3	
Student	395	32.7	31.3	4.1	
**Household gross income (AED/month)**					
< 5000	222	18.4	29.3	4.3	** < 0.001**^**d**^
5000- < 10,000	220	18.2	30.5	3.7	
10,000- < 20,000	249	20.6	31.0	3.8	
20,000- < 30,000	262	21.7	32.0	3.5	
30,000 and above	256	21.2	30.8	4.2	
**BMI Categories (kg/m**^**2**^**)**					
Underweight	54	4.5	30.5	3.9	**0.033**
Normal	577	47.7	31.1	3.9	
Overweight	389	32.2	30.6	3.9	
Obese	189	15.6	30.2	4.3	

^*^Based on Independent T-test and One-Way ANOVA at a 5% significance level

^a^Significant difference between third and first and second groups

^b^Significant difference between second and third and fourth groups

^c^Significant difference between first and third group

^d^Significant difference between first and all other groups

^e^Significant difference between second and fourth group

Table 1 also revealed that perceptions of healthy food varied significantly in most variables. For instance, differences based on sex were evident, with females tending to have a slightly more positive perception of a healthy diet than males (*p* = 0.002). Regarding age, significant differences were also observed (*p* < 0.001), with younger age groups (18–24 and 25–29) showing more positive perceptions of healthy eating compared to older age groups (30–39 and 40–65). Moreover, single participants tended to have a more positive perception of a healthy diet than married participants (*p* = 0.001).

Educational disparities are similarly apparent, with statistically significant differences between education levels, showcasing higher perception scores for those with postgraduate educational attainment than those with a diploma/college degree (p < 0.001). Moreover, significant differences were observed according to employment status, where students showed a higher perception of healthy eating than employed individuals (p = 0.001). In addition, significant differences were observed based on household income (*p* < 0.001), where higher income brackets were linked with a higher perception of a healthy diet. Based on BMI, significant differences were observed between those categorized as having normal weight and those as obese (*p* = 0.033). The differences in mean perception scores for each perception item are presented in Table S2 (Additional file 3).

### Sources of information

**Fig. 1:**
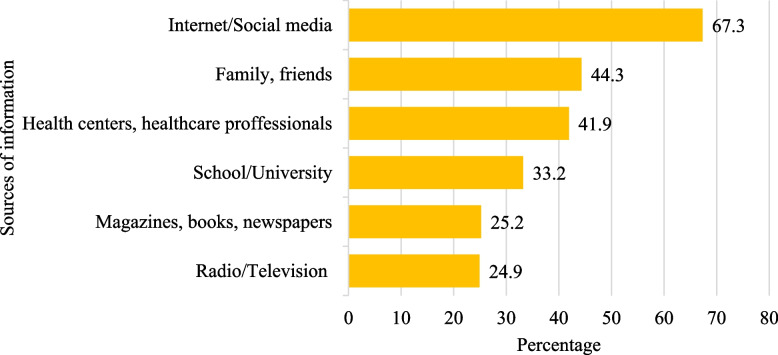
Sources of information about healthy eating (*n* = 1209) (percentage of those who responded “often” and “always”)

The participants were asked to rate how frequently they received information regarding consuming a healthy diet from various sources. Fig. 1 depicts the percentage of participants who use the sources more frequently. As per the participants, the internet/social media was the most common source of information (67.3%), followed by family/friends (44.3%), and health centres/dieticians (41.9%).

### Perception of healthy eating

**Fig. 2:**
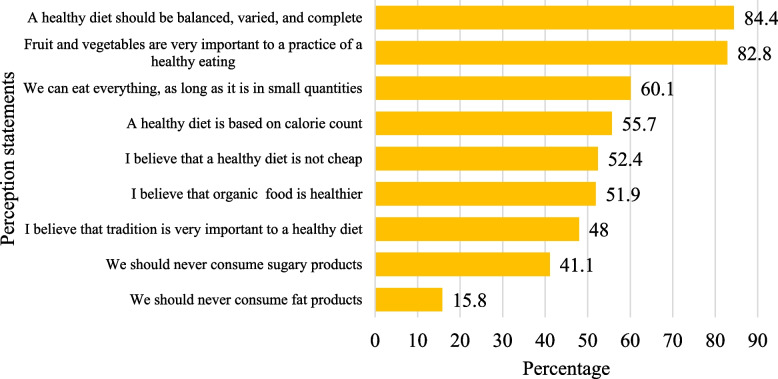
Perceptions of healthy eating (*n* = 1209) (percentage of those who responded “agree” and “strongly agree”)

Participants were asked to rate their agreement on different statements related to healthy eating based on a five-point Likert scale ranging from (1) strongly disagree to (5) strongly agree. Fig. 2 illustrates the percentage of participants whore responded “agree” or “strongly disagree” on the nine statements. Participants mostly agreed that a healthy diet should be balanced, varied, and complete (84.4%), that fruit and vegetables are essential to a practice of healthy eating (82.8%), and that they can eat everything as long as it is in small quantities (60.1%). Around half of the participants agreed that a healthy diet is based on calorie count (55.7%), and is not cheap (52.4%), and that organic food is healthier than normal food (51.9%). A lesser proportion of participants agreed that sugary and fatty products should be completely avoided (41.1% and 15.8%), respectively. The mean and SD for participants' responses to the nine statements are available in Table S1 (Additional file 2).

### Food choice motivations

**Fig. 3:**
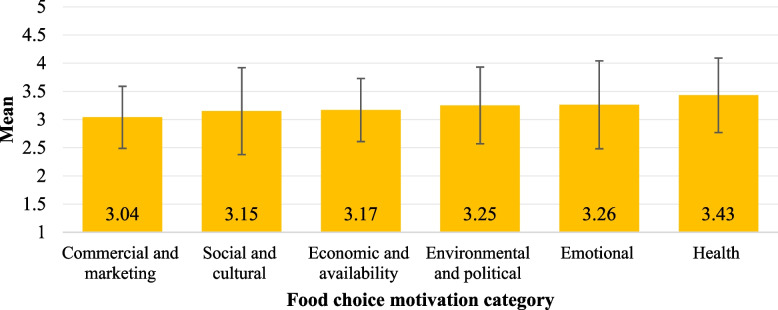
Mean food choice motivation scores for the study participants (range 1–5)

Figure 3 illustrates the study participants' mean food choice motivation scores (range 1–5). Health motivation (mean = 3.43, SD = 0.78) exhibited the greatest influence on the participants’ food choices, followed by emotional (mean = 3.26, SD = 0.68) and environmental and political motivations (mean = 3.25, SD = 0.66). In contrast, commercial and marketing motivation (mean = 3.04, SD = 0.56) had the least influence on the participants' food choices.

Eighteen out of 49 items had a median of four or above, corresponding to participants agreeing or strongly agreeing with these statements (Table 2). A variety of motivations guided the participants' food choices. Regarding health motivation, the participants expressed an inclination to agree with statements related to consuming food conducive to good health, including vitamins, minerals, and concerns about food safety: “It is important for me to eat food that keeps me healthy”, “It is important for me that my daily diet contains a lot of vitamins and minerals”, and “I am very concerned about the hygiene and safety of the food I eat”. They also showed concerns about the environment, specifically minimizing food waste when cooking and supporting animal rights in food production, showing their worries about the environment.

**Table 2:** The most important food choice motivations for the study participants ^a^ (*n* = 1,209)

Food choice motivation category	Statement	Mean	SD
**Health Motivation**	It is important for me to eat food that keeps me healthy	3.8	0.9
	It is important for me that my daily diet contains a lot of vitamins and minerals	3.7	0.9
	I am very concerned about the hygiene and safety of the food I eat	3.6	1.1
	I try to eat foods that do not contain additives	3.4	1
	There are some foods that I consume regularly, even if they may raise my cholesterol	3.3	1.1
	I avoid eating processed foods, because of their lower nutritional quality	3.3	1.1
**Emotional Motivation**	Food makes me feel good	3.8	1
	I often consume foods that help me relax (such as some teas, and herbal drinks)	3.4	1.1
	Food helps me cope with stress	3.3	1.1
**Economic and Availability Motivation**	I buy fresh vegetables to cook myself more often than frozen	3.7	1.1
	I usually choose food that has a good quality/price ratio	3.5	1
**Social and Cultural Motivations**	Meals are a time of fellowship and pleasure	3.7	1.0
	I eat more than usual when I have company	3.4	1.1
	I like to try new foods to which I am not accustomed	3.3	1.1
**Environmental and Political Motivation**	When I cook I have in mind the quantities to avoid food waste	3.9	1.0
	I prefer to eat food that has been produced in a way that animals' rights have been respected	3.4	1.1
**Marketing and Commercials Motivation**	When I go shopping I prefer to read food labels instead of believing in advertising campaigns	3.5	1.1
	Brands are important to me when making food choices	3.4	1.1

^a^Based on a median of 4 and above (agree and strongly agree)

Participants also considered emotional ties to food, economic factors, and social and cultural influences. They emphasized the emotional satisfaction derived from food; “Food makes me feel good”, balanced quality against price; “I buy fresh vegetables to cook myself more often than frozen” and “I usually choose food that has a good quality/price ratio”, and valued the social experience of eating “Meals are a time of fellowship and pleasure”. Their inclination towards reading food labels over-relying on marketing campaigns further demonstrated a discerning and informed approach to their food choices. Nonetheless, they were inclined to occasionally consume items that might impact cholesterol levels and the importance of brands in their food-related decision-making process. The mean food choice motivation for each item among study participants is displayed in Table S3 (Additional file 4).

Table 3 presents the differences in different food choice motivation categories across participants’ characteristics. Looking at sex-based disparities, females tended to prioritize emotional, environmental, and political motives significantly more than males (*p* < 0.001 and *p* = 0.002), respectively. Regarding age groups, significant differences were observed for all food choice motivation categories except for the environmental and political (*p* < 0.05). Individuals aged 40 and above exhibited significantly higher scores in health-related motivation and social and cultural motivations than younger age groups (*p* < 0.001 and *p* = 0.049), respectively. On the other hand, younger participants exhibited significantly higher scores concerning emotional, economic, and marketing motivations (*p* = 0.018. *p* = 0.002, and 0.007), respectively. In addition, significant differences were observed across different nationalities regarding economic and marketing motivations (*p* = 0.005 and *p* < 0.001), respectively. Differences based on marital status were also observed for all categories but marketing, where married participants leaned more toward health, social and cultural, and environmental and political motivations (*p* < 0.001, *p* = 0.010, and *p* = 0.018), respectively. According to education level, a gradual increase in health-related scores was observed, with the highest score among those with higher education degrees (*p* < 0.001). Moreover, employment status revealed differences in health motivation scores, social and cultural scores, and marketing scores, where students exhibited lower scores than their counterparts (*p* = 0.027, *p* = 0.034, and *p* = 0.012). On the other hand, they had significantly higher economic and availability scores (*p* = 0.010). Moreover, a gradual increase in health-related motivation scores was observed with rising income levels. Participants with higher household incomes, especially those in the 20,000–30,000 AED/month group, tend to prioritize health-related motives significantly more than those in lower-income brackets (*p*-values < 0.001). In contrast, significantly lower scores were observed with a higher income level regarding economic and marketing motivations (*p* = 0.008 and *p* < 0.001), respectively. Furthermore, emotional motivation score was the only category that differed significantly based on BMI. A significantly lower score was observed among underweight participants than their counterparts (*p* = 0.022).

**Table 3:** Differences in food choice motivation score based on participants’ socio-demographic characteristics (n = 1209)

Characteristics	Health		Emotional		Economic/ availability		Social/ cultural		Environmental/ political		Marketing/ commercials	
	*Mean*	SD	*Mean*	SD	*Mean*	SD	*Mean*	SD	*Mean*	SD	*Mean*	SD
**Sex**												
Female	3.40	0.60	3.37	0.75	3.18	0.66	3.16	0.56	3.36	0.71	3.04	0.55
Male	3.47	0.72	3.14	0.79	3.15	0.70	3.15	0.57	3.22	0.83	3.04	0.56
p-value *	0.061		< 0.001		0.489		0.783		0.002		0.802	
**Age (years)**												
18–24	3.34	0.66	3.29	0.80	3.24	0.69	3.11	0.59	3.25	0.79	3.03	0.54
25–29	3.42	0.65	3.39	0.74	3.21	0.70	3.19	0.54	3.30	0.71	3.17	0.58
30–39	3.43	0.76	3.22	0.87	3.12	0.74	3.14	0.64	3.31	0.83	3.04	0.52
40—65	3.58	0.54	3.17	0.69	3.06	0.60	3.21	0.48	3.36	0.72	2.99	0.57
***p-value***	< 0.001		0.018		0.002		0.049		0.248		0.007	
**Nationality**												
GCC countries	3.45	0.61	3.27	0.79	3.08	0.68	3.11	0.56	3.25	0.80	2.99	0.54
Arab (other countries)	3.40	0.68	3.27	0.79	3.18	0.68	3.16	0.57	3.31	0.73	3.10	0.56
Non-Arab	3.50	0.64	3.20	0.71	3.28	0.65	3.18	0.53	3.33	0.87	2.89	0.52
***p-value***	0.199		0.537		0.005		0.338		0.443		< 0.001	
**Marital status**												
Single	3.37	0.67	3.30	0.79	3.21	0.69	3.12	0.59	3.26	0.77	3.05	0.55
Married	3.53	0.62	3.20	0.75	3.10	0.66	3.20	0.53	3.36	0.77	3.02	0.55
***p-value***	** < 0.001**		**0.021**		**0.006**		**0.010**		**0.018**		0.252	
**Education level**												
High school or below	3.33	0.76	3.32	0.81	3.16	0.71	3.14	0.63	3.37	0.80	3.01	0.50
College certificate or Diploma	3.34	0.75	3.10	0.85	3.10	0.80	3.13	0.68	3.23	0.92	2.99	0.54
Bachelor’s degree	3.46	0.58	3.29	0.74	3.19	0.64	3.16	0.52	3.28	0.72	3.05	0.55
Postgraduate education	3.62	0.60	3.26	0.74	3.18	0.65	3.15	0.41	3.34	0.69	3.10	0.66
***p-value***	** < 0.001**		**0.018**		0.438		0.855		0.263		0.304	
**Employment status**												
Employed	3.47	0.70	3.23	0.78	3.15	0.68	3.16	0.57	3.28	0.79	3.09	0.57
Unemployed	3.45	0.56	3.25	0.77	3.08	0.68	3.21	0.54	3.39	0.77	2.97	0.54
Student	3.36	0.63	3.33	0.77	3.24	0.67	3.10	0.56	3.27	0.74	3.01	0.53
***p-value***	**0.027**		0.137		**0.010**		**0.034**		0.149		**0.012**	
**Household gross income level (AED/month)**												
< 5000	3.26	0.86	3.15	0.92	3.17	0.76	3.11	0.65	3.30	0.90	3.00	0.47
5000- < 10,000	3.44	0.58	3.34	0.76	3.21	0.70	3.19	0.57	3.39	0.72	3.00	0.50
10,000- < 20,000	3.48	0.57	3.26	0.72	3.18	0.62	3.20	0.53	3.30	0.73	3.02	0.53
20,000- < 30,000	3.58	0.53	3.35	0.67	3.25	0.56	3.16	0.49	3.32	0.68	3.24	0.64
30,000 and above	3.37	0.67	3.20	0.80	3.04	0.73	3.08	0.58	3.18	0.79	2.92	0.52
***p-value***	** < 0.001**		**0.018**		**0.008**		0.089		0.056		** < 0.001**	
**BMI Categories (kg/m**^**2**^**)**												
Underweight	3.28	0.76	2.95	0.93	3.16	0.71	3.06	0.60	3.34	0.84	2.97	0.48
Normal	3.44	0.66	3.29	0.79	3.20	0.69	3.14	0.56	3.30	0.77	3.08	0.58
Overweight	3.47	0.61	3.25	0.74	3.16	0.63	3.16	0.52	3.29	0.72	3.04	0.54
Obese	3.38	0.67	3.27	0.76	3.11	0.71	3.19	0.65	3.29	0.86	2.96	0.49
***p-value***	0.150		**0.022**		0.407		0.425		0.973		0.054	

^*^Based on Independent T-test and One-Way ANOVA at a 5% significance level

### Consumer segmentation

**Fig. 4:**
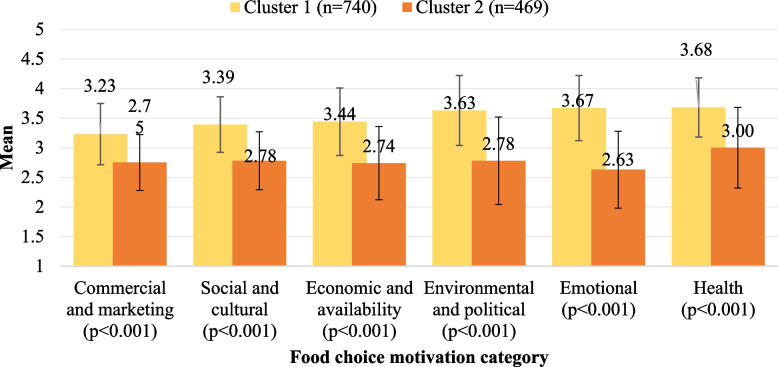
Mean food choice motivation scores for based on identified clusters (range 1–5)

Figure 4 presents the mean food choice motivation scores based on the results of the K-means cluster analysis, which identified two main clusters. The first cluster included most participants (*n* = 740, 61.2%), while the second cluster included about a third of the sample (*n* = 469, 38.2%). The analysis revealed that health-related motivations mostly influenced food choices among participants in both clusters. However, while Cluster 1 records a mean score of 3.68 for health, Cluster 2 follows closely behind with a significantly lower mean score of 3.00 (*p* < 0.001), suggesting that health considerations are relatively important for both clusters but notably more emphasized in Cluster 1.

Following health motivations, Cluster 1 demonstrates significantly higher mean scores in all other categories compared to Cluster 2. For instance, emotional factors represent the second highest mean score in Cluster 1 (mean = 3.67), whereas Cluster 2 ranks the lowest with a mean score of 2.63. This substantial difference indicates that emotional influences can exert a more considerable influence over food choices in Cluster 1 compared to Cluster 2. Moreover, environmental and political considerations exhibit the third highest mean score in Cluster 1 (3.63), significantly surpassing Cluster 2's mean score of 2.78 in the same category, indicating a stronger inclination towards environmental and political aspects influencing food choices within Cluster 1. Overall, the analysis reveals that participants in Cluster 1’s food choices are more influenced by health, emotional, environmental, and political motivations, while in Cluster 2, it is mainly health motivations that affect participants' choices.

### Consumer profiling

Table 4 shows the sociodemographic profiling in each cluster. A Pearson’s Chi-square test assessed the differences between the two clusters. Overall, significant associations with the clusters were observed for sex (*p* < 0.001) and income (*p* < 0.001). Comprising 61.2% of the total participants, Cluster 1 encompasses more female participants than males. A larger portion of this cluster falls within the 20,000—< 30,000 AED income brackets. On the other hand, Cluster 2, constituting 38.2% of the total participants, comprises a more significant proportion of males. It includes more individuals falling within higher income brackets (≥ 30,000 AED).

**Table 4:** Sociodemographic characteristics of study participants based on clusters (*n* = 1,209)

Characteristics	Total		Cluster 1		Cluster 2		*p*-value*
	***n***	***%***	***n***	**%**	***n***	**%**	
**Total**			740	61.2	469	38.2	
**Sex**							
Female	648	53.6	431	58.2	217	46.3	** < 0.001**
Male	561	46.4	309	41.8	252	53.7	
**Age (years)**							
18–24	527	43.6	321	43.4	206	43.9	0.455
25–29	172	14.2	114	15.4	58	12.4	
30–39	204	16.9	119	16.1	85	18.1	
40—65	306	25.3	186	25.1	120	25.6	
**Nationality**							
GCC countries	337	27.9	200	27.0	137	29.2	0.711
Arab (other countries)	714	59.1	442	59.7	272	58.0	
Non-Arab	158	13.1	98	13.2	60	12.8	
**Marital status**							
Single	741	61.3	458	61.9	283	60.3	0.590
Married	468	38.7	282	38.1	186	39.7	
**Education level**							
High school or below	239	19.8	153	20.7	86	18.3	0.079
College certificate or Diploma	187	15.5	99	13.4	88	18.8	
Bachelor’s degree	672	55.6	421	56.9	251	53.5	
Postgraduate education	111	9.2	67	9.1	44	9.4	
**Employment status**							
Employed	594	49.1	364	49.2	230	49.0	0.995
Unemployed	220	18.2	135	18.2	85	18.1	
Student	395	32.7	241	32.6	154	32.8	
**Household gross income level (AED/month)**							
< 5000	222	18.4	122	16.5	100	21.3	** < 0.001**
5000- < 10,000	220	18.2	136	18.4	84	17.9	
10,000- < 20,000	249	20.6	154	20.8	95	20.3	
20,000- < 30,000	262	21.7	189	25.5	73	15.6	
30,000 and above	256	21.2	139	18.8	117	24.9	
**BMI Categories (kg/m**^**2**^**)**							
Underweight	54	4.5	28	3.8	26	5.5	0.288
Normal	577	47.7	365	49.3	212	45.2	
Overweight	389	32.2	230	31.1	159	33.9	
Obese	189	15.6	117	15.8	72	15.4	

^*^Based on Pearson’s chi-square analysis at a 5% level of significance

## Discussion

This study explored perceptions of healthy eating and food choice motivations among a sample of adults in the UAE. It also segmented participants based on their food choice motivations to allow for tailored interventions and communication strategies to promote healthier dietary habits.

The present study highlighted the prevalence of the internet and social media as primary sources of information regarding healthy eating, mirroring previous research in the UAE [33]. Moreover, this reliance on digital platforms echoes global trends where these mediums have become essential for people to obtain health and nutrition-related content. For instance, in a study among Italian consumers, the Internet was the most frequently used source of information, followed by printed material [29]. While the digitalization of information seeking and sharing can be beneficial and more convenient, special attention should be paid to information quality and credibility, especially in terms of health and nutrition [34].

Research shows that more health-conscious individuals tend to assess the credibility of information based on the author's expertise, while less health-conscious individuals tend to rely more on the author's motivation and friendliness as credibility indicators [35]. Moreover, the information presented to people on these platforms may not always be what they are looking for, as social media and online platforms' algorithms rely on previous actions and interests to suggest content, resulting in unintentional exposure [36]. This kind of exposure can be harmful, especially to low-quality health or nutrition information. In particular, this is concerning in cases of nutrition misinformation, as research points out how these platforms can significantly impact eating behaviour and people’s food choices [37]. Thus, it is essential to enhance the digital literacy of the public. Educational initiatives that focus on teaching users how to critically evaluate the credibility of online content based on reliable indicators, such as the author's credentials and the evidence supporting the information, can be beneficial. Additionally, developing and promoting tools that help users identify and filter out low-quality information could mitigate the potential negative impact of algorithm-driven content exposure on eating behaviors and food choices.

Concerning perceptions of healthy eating, most individuals expressed their agreement for holistic approaches to maintaining a nutritious diet, emphasizing the importance of balanced meals, including fruits and vegetables, and the need for moderation in eating habits. In addition, most disagreed with the statement regarding abstaining from fat products. This is in line with research among Italian consumers, where such perceptions were mostly important to participants [29]. Moreover, they were in line with international guiding principles of healthy and sustainable diets [1, 38]. Half of the participants disagreed with the idea of monitoring caloric intake, while others agreed with it. Similarly, the participants had differing opinions regarding the affordability of maintaining a nutritious diet. The conflicting viewpoints highlighted gaps in comprehension and challenges in implementing theoretical concepts in practical situations. Educational programs should focus on clarifying the importance of balanced nutrition, including calorie monitoring, encourage mindful consideration of food groups and their proportions, and addressing the practical challenges of sustaining an affordable and nutritious diet.

The present study revealed significant demographic variations in perceptions of a healthy diet. Females had a slightly more positive perception than males, aligning with existing literature on gender differences in dietary attitudes and practices [39, 40]. Young adults also held more positive perceptions than older age groups, reflecting possible generational differences in health consciousness. Higher educational attainment was associated with more positive perceptions, echoing the impact of education on health-seeking behaviour and attitude [41]. Notably, those with normal weight had more positive perceptions than those classified as obese, reflecting possible challenges faced by individuals with higher BMI in adopting healthy dietary habits [29]. Overall, our findings align with other studies among Brazilian and Polish adults [42, 43], emphasizing the importance of considering these differences in establishing targeted interventions to improve overall healthy eating attitudes and habits among people.

Health motivation emerged as the predominant factor driving food choices, underscoring a more health-orientated behaviour among the study sample. This agrees with other studies among different populations in the Mediterranean countries [44], Italian [29], and Croatian consumers [45]. Moreover, emotional and environmental/political motivations also held significance among our participants, aligning with studies emphasizing emotional connections to food and growing concerns about sustainability and ethical aspects of consumption [46–48]. In addition, these findings agree with previous research on food choice motivation being the second most important motive for adults [29, 45]. Therefore, the prominence of health motivation in influencing food choices can be understood in the context of the UAE's comprehensive public health initiatives aimed at reducing the prevalence of chronic diseases such as obesity and diabetes. These campaigns likely increase public awareness and concern for health, reinforcing the significance of health-related food choices.

Upon examining individual items, the study highlighted key priorities among our participants. Participants mostly agreed on the importance of eating food that supports health, highlighting a shared value among participants on how good nutrition supports well-being. Moreover, they mostly agreed that food has an emotional significance for them and makes them feel good. This indicates that individuals have an emotional and psychological connection with food that goes beyond its nutritional value. Additionally, participants recognized meals as a time of fellowship and pleasure, which aligns with cultural and social norms in the UAE and the region [49]. Further, taking an active approach to reducing food waste while cooking was also important to them, indicating an increasing awareness and dedication to sustainability given the country’s efforts in achieving the United Nations Sustainable Development Goals, particularly zero hunger [48, 50]. The observed influence of environmental and political motivations may be attributed to the UAE's recent emphasis on sustainability, such as the National Food Security Strategy 2051. These motivations suggest a growing awareness of the environmental impact of food choices, which could be leveraged in future public health and sustainability campaigns.

Differences in food choice motivation scores based on sociodemographic factors, including gender, age, education, employment, and BMI, were observed in the present study. This aligns with studies focusing on determinants of food choices and acknowledging that certain personal factors such as biological features, physiological needs, cognitive factors, as well as sociocultural factors can play a pivotal role in people’s food choices [3, 51].

In the present study, distinct motivational patterns were outlined through segmentation via cluster analysis, which showed varying degrees of emphasis on motivations. Cluster 1, comprising 61.2% of the participants, showcased a comprehensive approach, emphasizing health first and focusing on emotional, environmental/political motivations. Cluster 2, conversely, demonstrated a health-focused approach with a relatively lesser inclination towards emotional and environmental factors, indicating a more concentrated emphasis on health considerations. These findings align with a study by Zwierczyk et al. [43], indicating that dietary behaviour is influenced by motivations that go beyond health considerations. While still significant, health motivations are closely intertwined with emotional, economic, and marketing motivations, pointing to a more holistic approach to understanding the factors that shape food choices among our participants, particularly in cluster 1 [43]. Additionally, our findings were comparable to a study among Italian consumers, where their cluster analysis identified groups influenced by health or emotional factors [29], and to a study among Croatian consumers, where the identified groups were influenced by emotional or health motivations [45]. The findings align with the Health Belief Model, which suggests that individuals are more likely to engage in health-promoting behaviors when they perceive a high risk of illness. In the UAE, awareness of diet-related health conditions may drive individuals to prioritize health when making food choices, especially given the country's high rates of non-communicable diseases.

Further, in the present study, some significant sociodemographic differences were observed based on clusters, especially in terms of sex and income. Cluster 1, which constituted the majority of participants, had a higher proportion of females. This could imply that women are more inclined to belong to a cluster that is influenced by a wide range of motivations, such as health, emotional, and environmental/political factors, compared to men. In contrast, Cluster 2, which had a larger percentage of males, was mainly focused on motivations related to health and considered other motivation categories less important. The composition of clusters based on sex could be explained by a range of factors such as societal norms, cultural influences, and individual preferences [3]. By comprehending these differences, we can develop focused interventions and communication tactics that meet the unique needs of specific gender groups, encouraging more sustainable and healthier food choices. In addition, the differences based on income levels indicate that financial factors may have an impact on determining the reasons behind food choices. People belonging to Cluster 1, who have lower incomes, may prioritize a variety of motivations, which could be influenced by affordability concerns. On the other hand, Cluster 2, who have higher incomes, may have the liberty to concentrate more on motivations related to health. This emphasizes the significance of considering socioeconomic status while devising interventions that cater to people with diverse economic backgrounds.

This study has several limitations. The use of an online survey may have excluded individuals without internet access, limiting the inclusivity of the sample. Additionally, the convenience sampling method may reduce the generalizability of the findings, particularly given the lower representation of non-Arab participants. Self-reported height and weight data used to calculate BMI may also introduce inaccuracies. Despite these limitations, the large and diverse sample size enhances the study's validity and provides valuable insights into food choice motivations in the UAE. The study is also noteworthy for its novelty in the country and the Middle East context, as it is the first to conduct research on this topic in the region. This not only significantly contributes to the existing body of knowledge but also addresses a notable gap in the literature.

## Conclusion

The findings of this study provide valuable insights into the motivations and perceptions influencing food choices among adults in the UAE. Health motivations emerged as the primary driver of food selection, with notable contributions from emotional and environmental considerations. These findings highlight the importance of addressing diverse motivators to promote healthier eating habits. Future interventions and policies should adopt a holistic approach, integrating health, emotional, economic, and environmental aspects to effectively encourage sustainable and nutritious dietary practices across different population groups.

## Supplementary Information

Supplementary Material 1.

Supplementary Material 2.

Supplementary Material 3.

Supplementary Material 4.

## Data Availability

The datasets used and/or analyzed during the current study are available from the corresponding author on reasonable request.

## Abbreviations

BMI: Body mass index
EATMOT: The Eating Motivation Scale
HCA: Hierarchical Cluster Analysis
I-CVI: Item-Level Content Validity Index
S-CVI/Ave: Scale-Level Content Validity Index
SD: Standard deviations
UAE: United Arab Emirates
UOS: University of Sharjah

## References

[1] Healthy diet [https://who.int/news-room/fact-sheets/detail/healthy-diet].

[2] Berry EM, Dernini S, Burlingame B, Meybeck A, Conforti P. Food security and sustainability: can one exist without the other? Public Health Nutr. 2015;18(13):2293–302. 25684016 10.1017/S136898001500021X PMC10271846

[3] Chen P-J, Antonelli M. Conceptual models of food choice: influential factors related to foods, individual differences, and society. Foods. 2020;9(12):1898. 33353240 10.3390/foods9121898 PMC7766596

[4] Jackson SE, Llewellyn CH, Smith L. The obesity epidemic–nature via nurture: a narrative review of high-income countries. SAGE Open Med. 2020;8:2050312120918265. 32435480 10.1177/2050312120918265 PMC7222649

[5] Djekic I, Bartkiene E, Szűcs V, Tarcea M, Klarin I, Černelić-Bizjak M, Isoldi K, El-Kenawy A, Ferreira V, Klava D, et al. Cultural dimensions associated with food choice: a survey based multi-country study. Int J Gastron Food Sci. 2021;26:100414.

[6] Organization WH: Diet, nutrition, and the prevention of chronic diseases: report of a joint WHO/FAO expert consultation, vol. 916: World Health Organization; 2003. 12768890

[7] Zobel EH, Hansen TW, Rossing P, von Scholten BJ. Global changes in food supply and the obesity epidemic. Curr Obes Rep. 2016;5(4):449–55. 27696237 10.1007/s13679-016-0233-8

[8] Hall KD. From dearth to excess: the rise of obesity in an ultra-processed food system. Philos Trans R Soc B. 1885;2023(378):20220214. 10.1098/rstb.2022.0214 PMC10363698 37482782

[9] Verde L, Frias-Toral E, Cardenas D. Editorial: environmental factors implicated in obesity. Front Nutr. 2023;10:1171507. 37215212 10.3389/fnut.2023.1171507 PMC10192899

[10] Al Amiri E, Abdullatif M, Abdulle A, Al Bitar N, Afandi EZ, Parish M, Darwiche G. The prevalence, risk factors, and screening measure for prediabetes and diabetes among Emirati overweight/obese children and adolescents. BMC Public Health. 2015;15(1):1–9. 26704130 10.1186/s12889-015-2649-6 PMC4690431

[11] Sobal J, Bisogni CA. Constructing food choice decisions. Ann Behav Med. 2009;38(suppl_1):s37–46. 19787306 10.1007/s12160-009-9124-5

[12] Rangel A. Regulation of dietary choice by the decision-making circuitry. Nat Neurosci. 2013;16(12):1717–24. 24270272 10.1038/nn.3561 PMC4053793

[13] Raquel PFG, Duarte J, Ferrão AC, Ferreira M, Correia P, Cardoso AP, Bartkiene E, Szűcs V, Nemes L, Ljubičić M, et al. The eating motivations scale (EATMOT): Development and validation by means of confirmatory factor analysis (CFA) and structural equation modelling (SEM). Slovenian J Public Health. 2021;60(1):4–9. 10.2478/sjph-2021-0002 PMC7780765 33488816

[14] Ferrão AC, Guiné RP, Correia P, Ferreira M, Duarte J, Lima J. Development of a questionnaire to assess people’s food choices determinants. Curr Nutr Food Sci. 2019;15(3):281–95.

[15] Guiné RPF, Bartkiene E, Szűcs V, Tarcea M, Ljubičić M, Černelič-Bizjak M, Isoldi K, EL-Kenawy A, Ferreira V, Straumite E et al: Study about food choice determinants according to six types of conditioning motivations in a sample of 11,960 participants. Foods 2020;9(7):888. 10.3390/foods9070888 PMC7404720 32645828

[16] Montanari Massimo. Food is culture: arts and traditions of the table: perspectives on culinary history. New York: Columbia University Press; 2006. p. 51–9.

[17] Hallström E, Carlsson-Kanyama A, Börjesson P. Environmental impact of dietary change: a systematic review. J Clean Prod. 2015;91:1–11.

[18] Gorski MT, Roberto CA. Public health policies to encourage healthy eating habits: recent perspectives. J Healthc Leadersh. 2015;7(null):81–90. 29355201 10.2147/JHL.S69188 PMC5740998

[19] National Nutrition Strategy 2030 [https://u.ae/en/about-the-uae/strategies-initiatives-and-awards/strategies-plans-and-visions/health/national-nutrition-strategy-2030].

[20] Al Sabbah H, Assaf EA, Al-Jawaldeh A, AlSammach AS, Madi H, Khamis Al Ali N, Al Dhaheri AS, Cheikh Ismail L. Nutrition situation analysis in the UAE: a review study. Nutrients. 2023;15(2):363. 10.3390/nu15020363 PMC9861891 36678240

[21] Ministry of Health launches 'School Lunch Box' programme to promote healthy lifestyles among children [https://www.wam.ae/en/details/1395302962802].

[22] UAE’s first online system to collect data about obesity among school students launched [https://wam.ae/en/details/1395302819727].

[23] What is Dubai fitness challenge? [https://www.dubaifitnesschallenge.com/about-us/].

[24] Hussain A, Elkelish WW, Al Mahameed M. Impact of excise tax on consumption, brand loyalty and health awareness: evidence from the United Arab Emirates. Cogent Bus Manag. 2023;10(1):2160579.

[25] 'Traffic light' nutrition labels on packaged food will not be mandatory [https://www.thenationalnews.com/uae/health/2021/12/12/traffic-light-nutrition-labels-on-packaged-food-no-longer-mandatory/].

[26] Mamdouh H, Hussain HY, Ibrahim GM, Alawadi F, Hassanein M, Al Zarooni A, Al Suwaidi H, Hassan A, Alsheikh-Ali A, Alnakhi WK. Prevalence and associated risk factors of overweight and obesity among adult population in Dubai: a population-based cross-sectional survey in Dubai, the United Arab Emirates. BMJ Open. 2023;13(1):e062053. 36693685 10.1136/bmjopen-2022-062053 PMC9884894

[27] Baniissa W, Radwan H, Rossiter R, Fakhry R, Al-Yateem N, Al-Shujairi A, Hasan S, Macridis S, Farghaly AA, Naing L. Prevalence and determinants of overweight/obesity among school-aged adolescents in the United Arab Emirates: a cross-sectional study of private and public schools. BMJ Open. 2020;10(12): e038667. 33310793 10.1136/bmjopen-2020-038667 PMC7735131

[28] Abduelkarem AR, Sharif SI, Bankessli FG, Kamal SA, Kulhasan NM, Hamrouni AM. Obesity and its associated risk factors among school-aged children in Sharjah, UAE. PLoS ONE. 2020;15(6):e0234244. 32502178 10.1371/journal.pone.0234244 PMC7274381

[29] Wongprawmas R, Mora C, Pellegrini N, Guiné RPF, Carini E, Sogari G, Vittadini E. Food choice determinants and perceptions of a healthy diet among Italian consumers. Foods. 2021;10(2):318. 33546323 10.3390/foods10020318 PMC7913531

[30] Wild D, Grove A, Martin M, Eremenco S, McElroy S, Verjee-Lorenz A, Erikson P. Principles of good practice for the translation and cultural adaptation process for patient-reported outcomes (PRO) measures: report of the ISPOR task force for translation and cultural adaptation. Value in health. 2005;8(2):94–104. 15804318 10.1111/j.1524-4733.2005.04054.x

[31] Salimian F. Measuring the return on investment of training modules of electrical protection and Uninterruptible Power Supply (UPS) using the corrective and AHP approaches. Math Probl Eng. 2021;2021:2635761.

[32] World Health Organization. Obesity: Preventing and managing the global epidemic. In. Geneva: World Health Organization; 2000. 11234459

[33] Awofeso N, Gaber Y, Bamidele M. Determinants of youth engagement with health information on social media platforms in United Arab Emirates. Health. 2019;11(02):249.

[34] Afful-Dadzie E, Afful-Dadzie A, Egala SB. Social media in health communication: a literature review of information quality. Health Inform Manag J. 2023;52(1):3–17. 10.1177/1833358321992683 33818176

[35] Kolarić A, Juric M, Peša Pavlović N: College Students’ Credibility Judgments on Healthy Diet Information on Social Media. In: Information Literacy in a Post-Truth Era: 2022// 2022; Cham: Springer International Publishing; 2022: 62–74.

[36] Kulshrestha J, Eslami M, Messias J, Zafar M, Ghosh S, Gummadi KP, Karahalios K: Quantifying Search Bias: Investigating Sources of Bias for Political Searches in Social Media. In: The 2017 ACM Conference. 2017: 417–432.

[37] Simeone M, Scarpato D. Sustainable consumption: how does social media affect food choices? J Clean Prod. 2020;277:124036.

[38] FAO and WHO: Sustainable healthy diets – Guiding principles. In*.* Rome, Italy: FAO and WHO; 2019.

[39] Lee J, Allen J. Gender differences in healthy and unhealthy food consumption and its relationship with depression in young adulthood. Community Ment Health J. 2021;57(5):898–909. 32602082 10.1007/s10597-020-00672-x

[40] Wu Y, Wang S, Shi M, Wang X, Liu H, Guo S, Tan L, Yang X, Wu X, Hao L. Awareness of nutrition and health knowledge and its influencing factors among Wuhan residents. Front Public Health. 2022;10:987755. 36276389 10.3389/fpubh.2022.987755 PMC9580461

[41] Cutler DM, Lleras-Muney A. Understanding differences in health behaviors by education. J Health Econ. 2010;29(1):1–28. 19963292 10.1016/j.jhealeco.2009.10.003 PMC2824018

[42] Ferreira VA, Lopes ACS, Guiné RdPF, Ribeiro MC, Pires ISC, Miranda LS, Magalhães R. Perception of healthy eating among adults participating in the Eat-Mot survey in Brazil. Res Soc Develop. 2021;10(2):e50110212601.

[43] Zwierczyk U, Sowada C, Duplaga M. Eating choices - the roles of motivation and health literacy: a cross-sectional study. Nutrients. 2022;14(19):4026. 36235678 10.3390/nu14194026 PMC9573739

[44] Guiné R, Ferrão AC, Ferreira M, Correia P, Cardoso AP, Duarte J, Rumbak I, Shehata A-M, Vittadini E, Papageorgiou M. The motivations that define eating patterns in some Mediterranean countries. Nutr Food Sci. 2019;49(6):1126–41.

[45] Ilić A, Rumbak I, Dizdarić D, Matek Sarić M, Colić Barić I, Guiné RPF. Motivations associated with food choices among adults from urban setting. Foods. 2023;12(19):3546. 37835199 10.3390/foods12193546 PMC10572751

[46] Ljubičić M, Matek Sarić M, Klarin I, Rumbak I, Colić Barić I, Ranilović J, Dželalija B, Sarić A, Nakić D, Djekic I, et al. Emotions and food consumption: emotional eating behavior in a European population. Foods. 2023;12(4):872. 36832947 10.3390/foods12040872 PMC9957014

[47] Lehikoinen E, Salonen AO. Food preferences in Finland: Sustainable diets and their differences between groups. Sustainability. 2019;11(5):1259.

[48] Hashim M, Ismail LC, Abbas N, Ali J, Saeed F, Mohamed A, Mashal A, Naja F. Sustainable diets among youth: Validity and reliability of a questionnaire assessing knowledge, attitudes, practices, and willingness to change. J Hum Nutr Diet. 2023;36:2280–94. 37282743 10.1111/jhn.13190

[49] Savvaidis IN, Al Katheeri A, Lim S-HE, Lai K-S, Abushelaibi A. Chapter 1 - Traditional foods, food safety practices, and food culture in the Middle East. In: Savvaidis IN, Osaili TM, editors. Food Safety in the Middle East. Cambridge: Academic Press; 2022. p. 1–31. 10.1016/B978-0-12-822417-5.00009-X.

[50] National Food Security Strategy 2051 [https://u.ae/en/about-the-uae/leaving-no-one-behind/2zerohunger].

[51] Adan RAH, Belot M, Brunstrom JM, Dickson SL, Hare T, Leng G, Maier S, Menzies J, Preissl H, Reisch LA, et al. The determinants of food choice. Proceed Nutr Soci. 2017;76(3):316–27. 10.1017/S002966511600286X 27903310

