# Supplementary material for: Food choice motivations and perceptions of healthy eating: a cross-sectional study among consumers in the UAE
Source: BMC Public Health. 2025 Feb 4;25:442. doi: 10.1186/s12889-024-20836-8 (PMC11792200; doi:10.1186/s12889-024-20836-8)
Supplement: Supplementary file 1 — Supplementary Material 1. [file 12889_2024_20836_MOESM1_ESM.pdf]

I. Socio-demographic information

1. What is your gender?
- Female
- Male

2. How old are you in years?
- ____________

3. Which emirate do you reside in?
- Abu Dhabi
- Dubai
- Sharjah
- Ajman
- Umm Al Quwain
- Ras Al Khaimah
- Fujairah

4. What is your nationality?
- Emirati citizen
- Arab (GCC: Bahrain, Kuwait, Oman, Qatar, Saudi Arabia)
- Arab (other countries)
- Non-Arab

5. What is your marital status?
- Single
- Married
- Divorced/Widowed

6. How many children do you have?
- Not applicable
- 0
- 1-2
- 3-4
- 5 or more

7. What is your education Level?
- Less than high school
- High School
- College/ Diploma
- University Degree
- Higher education masters/doctorate

8. What is your employment status?
- Full-time employment
- Part-time employment
- Unemployed
- Retired
- Self-employed
- Student (unemployed)

9. What is your household income level? (AED/month)
- <5000
- 5000- <10,000
- 10000- <20,000
- 20,000- <30,000
- 30,000 and above

10. What is your current weight? __________(kg)

11. What is your height? ___________ (cm)

II. Perceptions of healthy eating

What is your perception of a healthy diet? *Please indicate your opinion about the following statements*

Statement	Strongly disagree	Disagree	Neutral	Agree	Strongly agree
1. A healthy diet is based on calorie count					
2. We should never consume sugary products					
3. Fruit and vegetables are very important to a practice of a healthy eating					
4. A healthy diet should be balanced, varied, and complete					
5. We can eat everything, as long as it is in small quantities					
6. I believe that a healthy diet is not cheap					
7. In my opinion, it is strange that some people have cravings for sweets					
8. I believe that tradition is very important to a healthy diet					
9. I believe that organic food is healthier					
10. We should never consume fat products					

III. Sources of information about a healthy diet

Where do you usually find information about eating a healthy diet? *Please indicate with what frequency you find information about eating a healthy diet.*

Sources	Never	Rarely	Sometimes	Often	Always
1. Health centers, hospitals, general practitioners (doctors or dietitians)					
2. Radio/Television					
3. School/University					
4. Magazines, books, newspapers					
5. Internet/Social media					
6. Family, friends					

IV. Food choices motivation

Please indicate your opinion about the following statements.

1. Health Motivation

Statement	Strongly disagree	Disagree	Neutral	Agree	Strongly agree
1. I am very concerned about the hygiene and safety of the food I eat					
2. It is important for me that my diet is low in fat					
3. Usually I follow a healthy and balanced diet					
4. It is important for me that my daily diet contains a lot of vitamins and minerals					
5. There are some foods that I consume regularly, even if they may raise my cholesterol					
6. I try to eat foods that do not contain additives					
7. I avoid eating processed foods, because of their lower nutritional quality					
8. It is important for me to eat food that keeps me healthy					
9. There are some foods that I consume regularly, even if they may raise my blood glycemia					
10. I avoid foods with genetically modified organisms					

2. Emotional motivation

Statement	Strongly disagree	Disagree	Neutral	Agree	Strongly agree
1. Food helps me cope with stress					
2. I usually eat food that helps me control my weight					
3. I often consume foods that keep me awake and alert (such as coffee, coke, and energy drinks)					
4. I often consume foods that help me relax (such as some teas, herbal drinks)					
5. Food makes me feel good					
6. When I feel lonely, I console myself by eating					
7. I eat more when I have nothing to do					
8. For me, food serves as an emotional consolation					
9. I have more cravings for sweets when I am depressed					

3. Economic and availability motivations

Statement	Strongly disagree	Disagree	Neutral	Agree	Strongly agree
1. I usually choose food that has a good quality/price ratio					
2. The main reason for choosing a food is its low price					
3. I choose the food I consume because it is convenient to purchase					
4. I buy fresh vegetables to cook myself more often than frozen					
5. I usually buy food that is easy to prepare					
6. I usually buy food that is on sale					
7. I prefer to buy food that is ready-to-eat or pre-cooked					

4. Social & cultural motivations

Statement	Strongly disagree	Disagree	Neutral	Agree	Strongly agree
1. Meals are a time of fellowship and pleasure					
2. I eat more than usual when I have company					
3. It is important to me that the food I eat is similar to the food I ate when I was a child					
4. I eat certain foods because other people (my colleagues, friends, family) also eat it					
5. I prefer to eat alone					
6. I choose the foods I eat, because it fits the season					
7. I eat certain foods because I am expected to eat them					
8. I like to try new foods to which I am not accustomed					
9. I usually eat food that is trendy					

5. Environmental and political motivation

Statement	Strongly disagree	Disagree	Neutral	Agree	Strongly agree
1. It is important to me that the food I eat is prepared/packed in an environmentally friendly way					
2. When I cook I have in mind the quantities to avoid food waste					
3. It is important to me that the food I eat comes from my own country					
4. I prefer to eat food that has been produced in a way that animals' rights have been respected					
5. I choose foods that have been produced in countries where human rights are not violated					
6. I avoid going to restaurants that do not have a recovery policy of food surplus					
7. I prefer to buy foods that comply with policies of minimal usage of packaging					

6. Marketing and commercials motivation

Statement	Strongly disagree	Disagree	Neutral	Agree	Strongly agree
1. When I buy food I usually do not care about the marketing campaigns happening in the shop					
2. I eat what I eat because I recognize it from advertisements or have seen it on TV					
3. I usually buy food that spontaneously appeals to me (e.g. situated at eye level, appealing colors, pleasant packaging)					
4. When I go shopping I prefer to read food labels instead of believing in advertising campaigns					
5. Food advertising campaigns increase my desire to eat certain foods					
6. Brands are important to me when making food choices					
7. I try to schedule my food shopping for when I know there are promotions or discounts					
