# Supplementary material for: Food choice motivations and perceptions of healthy eating: a cross-sectional study among consumers in the UAE
Source: BMC Public Health. 2025 Feb 4;25:442. doi: 10.1186/s12889-024-20836-8 (PMC11792200; doi:10.1186/s12889-024-20836-8)
Supplement: Supplementary file 2 — Supplementary Material 2. [file 12889_2024_20836_MOESM2_ESM.pdf]

Table S1. Perceptions of healthy eating among study participants (*n* =1,209)

Statement		Mean	SD
P.1	A healthy diet should be balanced, varied, and complete	4.1	1.0
P.2	Fruit and vegetables are very important to a practice of a healthy eating	4.0	1.1
P.3	A healthy diet is based on calorie count	3.5	1.1
P.4	We can eat everything, as long as it is in small quantities	3.4	1.1
P.5	I believe that organic food is healthier	3.4	1.1
P.6	I believe that a healthy diet is not cheap	3.3	1.2
P.7	I believe that tradition is very important to a healthy diet	3.2	1.2
P.8	We should never consume sugary products	3.0	1.2
P.9	We should never consume fat products	2.4	1.1
