# Supplementary material for: Food choice motivations and perceptions of healthy eating: a cross-sectional study among consumers in the UAE
Source: BMC Public Health. 2025 Feb 4;25:442. doi: 10.1186/s12889-024-20836-8 (PMC11792200; doi:10.1186/s12889-024-20836-8)
Supplement: Supplementary file 3 — Supplementary Material 3. [file 12889_2024_20836_MOESM3_ESM.pdf]

Table S2. Differences in perceptions of healthy diet based on participants' socio-demographic characteristics (n=1209)

Characteristics	P1		P2		P3		P4		P5		P6		P7		P8		P9	
	Mean	SD	Mean	SD	Mean	SD	Mean	SD	Mean	SD	Mean	SD	Mean	SD	Mean	SD	Mean	SD
**Sex**																		
Female	4.23	0.96	4.11	0.98	3.27	1.10	3.59	1.07	3.35	1.04	3.30	1.16	3.16	1.14	2.93	1.19	2.26	1.00
Male	4.04	1.05	3.97	1.12	3.47	1.15	3.40	1.15	3.39	1.14	3.32	1.19	3.31	1.29	3.16	1.24	2.55	1.16
**p-value ***	0.001		0.020		0.002		0.003		0.520		0.694		0.033		0.001		<0.001	
**Age (years)**																		
18-24	4.23	1.04	4.10	1.08	3.15	1.19	3.43	1.10	3.23	1.07	3.35	1.20	3.05	1.20	2.69	1.17	2.18	1.08
25-29	4.17	0.93	4.04	1.01	3.59	1.02	3.51	1.13	3.43	1.03	3.48	1.07	3.29	1.18	2.96	1.21	2.47	1.03
30-39	3.97	1.11	3.97	1.14	3.35	1.23	3.43	1.26	3.43	1.20	3.17	1.29	3.23	1.27	3.29	1.25	2.72	1.16
40+	4.09	0.89	3.99	0.94	3.61	0.93	3.67	1.01	3.54	1.05	3.23	1.08	3.49	1.17	3.50	1.10	2.50	1.01
**p-value**	0.012		0.384		0.014		<0.001		0.001		0.032		<0.001		<0.001		<0.001	
**Nationality**																		
GCC countries	4.15	1.00	4.04	1.03	3.43	1.11	3.48	1.09	3.38	1.14	3.24	1.21	3.24	1.22	3.28	1.20	2.44	1.08
Arab (other countries)	4.10	0.99	4.01	1.03	3.36	1.14	3.55	1.11	3.35	1.05	3.34	1.15	3.22	1.21	2.98	1.22	2.37	1.06
Non-Arab	4.28	1.05	4.16	1.14	3.22	1.16	3.34	1.18	3.46	1.12	3.33	1.22	3.22	1.21	2.75	1.19	2.39	1.24
**p-value**	0.110		0.293		0.101		0.151		0.482		0.467		0.979		<0.001		0.580	
**Marital status**																		
Single	4.17	1.04	4.05	1.08	3.27	1.16	3.46	1.12	3.28	1.07	3.35	1.18	3.09	1.21	2.78	1.18	2.26	1.08
Married	4.09	0.94	4.02	0.99	3.51	1.06	3.57	1.09	3.51	1.09	3.24	1.15	3.45	1.19	3.44	1.17	2.60	1.08
**p-value**	0.148		0.591		<0.001		0.102		<0.001		0.105		<0.001		<0.001		<0.001	
**Education level**																		
High school or less	4.07	1.08	4.03	1.13	3.22	1.14	3.46	1.15	3.33	1.13	3.31	1.15	3.10	1.28	2.96	1.22	2.45	1.18
College/Diploma	3.97	1.19	3.87	1.23	3.07	1.33	3.29	1.27	3.27	1.19	3.18	1.23	3.08	1.24	3.01	1.27	2.49	1.15
Bachelor's degree	4.21	0.94	4.10	0.98	3.46	1.06	3.54	1.06	3.40	1.06	3.33	1.18	3.25	1.14	3.09	1.22	2.35	1.05
Higher education	4.14	0.84	4.00	0.92	3.55	1.04	3.68	0.99	3.48	0.97	3.38	1.06	3.59	1.37	2.92	1.13	2.34	1.01
**p-value**	0.017		0.062		0.010		<0.001		0.300		0.376		0.002		0.319		0.347	
**Employment status**																		
Employed	4.01	1.02	3.95	1.08	3.50	1.11	3.48	1.10	3.42	1.10	3.35	1.15	3.31	1.25	3.13	1.21	2.53	1.08
Unemployed	4.23	0.88	4.08	0.91	3.40	1.01	3.42	1.10	3.44	1.08	3.10	1.21	3.28	1.14	3.41	1.16	2.35	1.05
Student	4.28	1.02	4.16	1.05	3.13	1.19	3.70	1.12	3.26	1.06	3.37	1.18	3.07	1.18	2.70	1.18	2.21	1.09
**p-value**	<0.001		0.006		0.008		<0.001		0.042		0.013		0.007		<0.001		<0.001	
**Household income level (AED/month)**																		
<5000	3.84	1.19	3.73	1.33	3.12	1.23	3.26	1.23	3.13	1.18	3.28	1.26	2.98	1.20	3.11	1.24	2.64	1.21
5000- <10,000	4.13	1.05	4.06	1.01	3.60	1.06	3.51	1.12	3.51	1.07	3.40	1.16	3.18	1.25	3.27	1.15	2.58	1.03
10000- <20,000	4.27	0.86	4.18	0.94	3.40	1.08	3.59	1.07	3.43	1.05	3.36	1.16	3.20	1.17	3.14	1.20	2.41	1.10
20,000- <30,000	4.24	0.77	4.15	0.80	3.46	1.05	3.68	0.91	3.63	0.89	3.41	1.05	3.56	1.17	2.69	1.13	2.16	0.88
30,000 and above	4.18	1.09	4.05	1.08	3.23	1.18	3.43	1.18	3.14	1.16	3.11	1.21	3.16	1.23	3.02	1.29	2.23	1.15
**p-value**	<0.001		<0.001		<0.001		<0.001		<0.001		0.021		<0.001		<0.001		<0.001	
**BMI Categories (kg/m2)**																		
Underweight	4.20	1.11	4.11	1.14	3.06	1.12	3.22	1.11	3.24	1.10	3.24	1.18	2.89	1.16	2.65	1.18	2.31	1.02
Normal	4.20	0.95	4.05	1.02	3.27	1.15	3.54	1.10	3.34	1.07	3.34	1.16	3.20	1.21	2.84	1.21	2.32	1.08
Overweight	4.07	1.02	4.02	1.03	3.49	1.05	3.55	1.09	3.46	1.09	3.29	1.17	3.32	1.23	3.17	1.17	2.51	1.10
Obese	4.07	1.10	4.03	1.12	3.46	1.20	3.37	1.17	3.32	1.12	3.28	1.22	3.21	1.20	3.49	1.18	2.40	1.10
**p-value**	0.148		0.929		0.062		0.002		0.247		0.853		0.075		<0.001		0.063	

* Based on Independent T-test and One-Way ANOVA at a 5% significance level. P1: A healthy diet should be balanced, varied, and complete; P2: Fruit and vegetables are very important to a practice of healthy eating; P3: A healthy diet is based on calorie count; P4: We can eat everything, as long as it is in small quantities; P5: I believe that organic food is healthier; P6: I believe that a healthy diet is not cheap; P7: I believe that tradition is very important to a healthy diet; P8: We should never consume sugary products; P9: We should never consume fat products.
