# Supplementary material for: Food choice motivations and perceptions of healthy eating: a cross-sectional study among consumers in the UAE
Source: BMC Public Health. 2025 Feb 4;25:442. doi: 10.1186/s12889-024-20836-8 (PMC11792200; doi:10.1186/s12889-024-20836-8)
Supplement: Supplementary file 4 — Supplementary Material 4. [file 12889_2024_20836_MOESM4_ESM.pdf]

Table S3. Food choices motivation among study participants (n =1,209)

Statement	Mean	SD
**Health Motivation**	3.4	0.7
I am very concerned about the hygiene and safety of the food I eat	3.6	1.1
It is important for me that my diet is low in fat	3.3	1.0
Usually I follow a healthy and balanced diet	3.3	1.0
It is important for me that my daily diet contains a lot of vitamins and minerals	3.7	1.0
There are some foods that I consume regularly, even if they may raise my cholesterol	3.3	1.1
I try to eat foods that do not contain additives	3.4	1.0
I avoid eating processed foods, because of their lower nutritional quality	3.4	1.1
It is important for me to eat food that keeps me healthy	3.8	0.9
There are some foods that I consume regularly, even if they may raise my blood glycemia	3.3	1.1
I avoid foods with genetically modified organisms	3.3	1.1
**Emotional motivation**	3.3	0.8
Food helps me cope with stress	3.3	1.2
I usually eat food that helps me control my weight	3.3	1.0
I often consume foods that keep me awake and alert (such as coffee, coke, and energy drinks)	3.2	1.2
I often consume foods that help me relax (such as some teas, and herbal drinks)	3.4	1.1
Food makes me feel good	3.8	1.0
When I feel lonely, I console myself by eating	3.0	1.2
I eat more when I have nothing to do	3.1	1.2
For me, food serves as an emotional consolation	3.1	1.2
I have more cravings for sweets when I am depressed	3.2	1.3
**Economic and availability motivations**	3.2	0.7
I usually choose food that has a good quality/price ratio	3.5	1.0
The main reason for choosing a food is its low price	2.7	1.1
I choose the food I consume because it is convenient to purchase	3.3	1.1
I buy fresh vegetables to cook myself more often than frozen	3.7	1.1
I usually buy food that is easy to prepare	3.2	1.1
I usually buy food that is on sale	3.1	1.1
I prefer to buy food that is ready-to-eat or pre-cooked	2.7	1.2
**Social & cultural motivations**	3.2	0.6

Meals are a time of fellowship and pleasure	3.7	1.0
I eat more than usual when I have company	3.4	1.1
It is important to me that the food I eat is similar to the food I ate when I was a child	2.9	1.1
I eat certain foods because other people (my colleagues, friends, family) also eat it	2.9	1.1
I prefer to eat alone	2.7	1.2
I choose the foods I eat, because it fits the season	3.0	1.1
I eat certain foods because I am expected to eat them		
I like to try new foods to which I am not accustomed	2.8	1.1
I usually eat food that is trendy	3.3	1.1
**Environmental and political motivation**	3.3	0.8
It is important to me that the food I eat is prepared/packed in an environmentally friendly way	3.2	1.1
When I cook I have in mind the quantities to avoid food waste	3.9	1.0
It is important to me that the food I eat comes from my own country	3.0	1.1
I prefer to eat food that has been produced in a way that animals' rights have been respected	3.5	1.1
I choose foods that have been produced in countries where human rights are not violated	3.3	1.1
I avoid going to restaurants that do not have a recovery policy of food surplus	3.1	1.1
I prefer to buy foods that comply with policies of minimal usage of packaging	3.2	1.0
**Marketing and commercials motivation**	3.0	0.6
When I buy food I usually do not care about the marketing campaigns happening in the shop	3.2	1.1
I eat what I eat because I recognize it from advertisements or have seen it on TV	2.9	1.0
I usually buy food that spontaneously appeals to me (e.g. situated at eye level, appealing colors, pleasant packaging)	3.3	1.1
When I go shopping I prefer to read food labels instead of believing in advertising campaigns	3.5	1.1
Food advertising campaigns increase my desire to eat certain foods	3.2	1.1
Brands are important to me when making food choices	3.4	1.1
I try to schedule my food shopping for when I know there are promotions or discounts	3.2	1.2
